# Effects of an Eight Week Very Low-Calorie Ketogenic Diet (VLCKD) on White Blood Cell and Platelet Counts in Relation to Metabolic Dysfunction-Associated Steatotic Liver Disease (MASLD) in Subjects with Overweight and Obesity

**DOI:** 10.3390/nu15204468

**Published:** 2023-10-21

**Authors:** Sara De Nucci, Caterina Bonfiglio, Rosanna Donvito, Martina Di Chito, Nicole Cerabino, Roberta Rinaldi, Annamaria Sila, Endrit Shahini, Vito Giannuzzi, Pasqua Letizia Pesole, Sergio Coletta, Elsa Lanzilotta, Giuseppina Piazzolla, Raffaele Cozzolongo, Gianluigi Giannelli, Giovanni De Pergola

**Affiliations:** 1 Center of Nutrition for the Research and the Care of Obesity and Metabolic Diseases, National Institute of Gastroenterology IRCCS “Saverio de Bellis”, 70013 Castellana Grotte, Italyroberta.rinaldi@irccsdebellis.it (R.R.);; 2 Laboratory of Epidemiology and Statistics, National Institute of Gastroenterology “Saverio de Bellis”, IRCCS Hospital, Castellana Grotte, 70013 Bari, Italy; 3 Department of Gastroenterology, National Institute of Gastroenterology “Saverio de Bellis”, IRCCS Hospital, Castellana Grotte, 70013 Bari, Italy; endrit.shahini@irccsdebellis.it (E.S.);; 4 Core Facility Biobank, National Institute of Gastroenterology “Saverio de Bellis”, IRCCS Hospital, Castellana Grotte, 70013 Bari, Italy; 5 Laboratory of Clinical Pathology, National Institute of Gastroenterology “Saverio de Bellis”, IRCCS Hospital, Castellana Grotte, 70013 Bari, Italy; 6 Interdisciplinary Department of Medicine, School of Medicine, University of Bari “Aldo Moro”, Piazza Giulio Cesare 11, 70124 Bari, Italy; giuseppina.piazzolla@uniba.it; 7 Scientific Direction, National Institute of Gastroenterology “Saverio de Bellis”, IRCCS Hospital, Castellana Grotte, 70013 Bari, Italy; gianluigi.giannelli@irccsdebellis.it

**Keywords:** white blood cell (WBC), platelets (PLT), non-alcoholic fatty liver disease (NAFLD), metabolic dysfunction-associated steatotic liver disease (MASLD), very low-calorie ketogenic diet (VLCKD), obesity, insulin resistance, transient elastography (FibroScan)

## Abstract

Obesity and metabolic dysfunction-associated steatotic liver disease (MASLD) are frequently associated conditions characterized by low-grade inflammation. Very low-calorie ketogenic diet (VLCKD) strategies are commonly used to simultaneously obtain weight loss and an improvement of liver steatosis. We evaluated the efficacy of 8 weeks’ VLCKD in decreasing the white blood cell (WBC) and platelet (PLT) counts, as well as liver steatosis and fibrosis, diagnosed using transient elastography (FibroScan). Metabolic and anthropometric parameters commonly associated with MASLD were also evaluated. This study included 87 participants; 58 women and 29 men aged between 18 and 64 years with overweight (18%) or obesity (82%), but not taking any medication. Anthropometric measurements, bioimpedance analysis, and biochemical assays were performed before and after the dietary intervention. BMI (kg/m^2^) (*p*-value < 0.001), waist circumference (cm) (*p*-value < 0.001), and fat mass (kg) (*p*-value < 0.001) were significantly decreased following VLCKD. After VLCKD, the FibroScan parameter CAP (db/m), which measures the accumulation of fatty liver, significantly decreased (*p*-value < 0.001), as did liver stiffness (kPA), the FibroScan parameter quantifying liver fibrosis (*p*-value < 0.05). Seemingly, WBC (*p*-value < 0.001) and PLT (*p*-value < 0.001) counts were lowered by VLCKD in the whole group; however, the decrease in WBC and platelet counts were significant only in patients with steatosis (CAP ≥ 215 dB/m). Fasting blood glucose (*p*-value < 0.001), insulin (*p*-value < 0.001), HbA1c (*p*-value < 0.001), triglycerides (*p*-value < 0.001), total cholesterol (*p*-value < 0.001), LDL-cholesterol (*p*-value < 0.001), HDL-cholesterol (*p*-value < 0.001); γGT (*p*-value < 0.001) blood levels and insulin resistance (as measured by HOMAIR) (*p*-value < 0.001); and systolic (*p*-value < 0.001), and diastolic (*p*-value < 0.001) blood pressure levels, were all significantly lower after VLCKD. In contrast, blood levels of vitamin D were higher following the diet (*p*-value < 0.001). We conclude that treating subjects with overweight and obesity with VLCKD is followed by a simultaneous reduction in WBCs and platelets, the expression of low-grade inflammation, and of liver steatosis and fibrosis. Therefore, we can hypothesize that VLCKD decreases general and liver low-grade inflammation, thus improving liver health.

## 1. Background

Nowadays, fatty liver disease is the main cause of liver disease rather than viral hepatitis, probably as a result of the increasing prevalence of metabolic syndrome. Non-alcoholic fatty liver disease (NAFLD) is now the most common form of chronic liver disease in developed countries [1], accounting for more than 25% of cases among the adult population of Europe, where it affects up to 30% of people worldwide [2]. NAFLD is more frequent among individuals with metabolic disorders like obesity, type 2 diabetes, or metabolic syndrome, in which its prevalence exceeds 70% [3,4]. Inactivity, a diet rich in calories, and insulin resistance (IR) are the primary triggers of NAFLD [5], as well as exposure to environmental risk factors and background genetic factors [6]. The most significant risks associated with NAFLD are abdominal obesity, insulin resistance, type 2 diabetes mellitus, hypertriglyceridemia and low high-density lipoproteins (HDL), hyperuricemia, and a family history for type 2 diabetes [7,8,9]. Hepatic steatosis is the term used to describe hepatic fat deposition of 5% or more that is unconnected to drug consumption, viral infections, or excessive drinking of alcohol (30 g/day for males and 20 g/day for women) [10,11]. Hepatic steatosis can be differentiated from non-alcoholic steatohepatitis (NASH), a subtype of NAFLD in which fat accumulation is linked to inflammation of hepatocytes, with or without fibrosis [12], increasing the likelihood of evolution to liver cirrhosis and eventually hepatocellular carcinoma [13]. It is important to remember that NASH remains the second reason for liver transplantation in the US [14]. The American Association for the Study of Liver Diseases (AASLD) recently deleted the terms NAFLD and NASH because they are confounders and potentially stigmatizing [15]. Hence, the term steatotic liver disease (SLD) was chosen as an all-embracing term encompassing the various aetiologies of steatosis, and the term NAFLD was replaced by metabolic dysfunction-associated steatotic liver disease (MASLD), that includes the presence of at least one of the five cardiometabolic risk factors for metabolic syndrome [15]

Liver biopsy is the gold standard for diagnosing, staging, and managing patients with MASLD, but it is too invasive to be used frequently in clinical practice. Transient elastography (FibroScan), a well-known ultrasound-based method for clinical use, supports a reliable and thorough assessment of hepatic steatosis across various conditions [16,17]. The mainstays of MASLD prevention include lifestyle adjustments, a balanced diet, and increased physical exercise. These changes improve IR, decrease systemic inflammation, promote weight reduction, lower body fat, and increase skeletal muscle mass [18].

The inflammatory state that accompanies adiposity is called “low-grade” inflammation, due to an up-regulation of pro-inflammatory markers and down-regulation of anti-inflammatory cytokines [19]. Risk factors for MASLD also cause low-grade chronic inflammation, which contributes to liver disease progression towards cirrhosis [20].

Current recommendations indicate that the most effective treatment for inflammation is weight reduction, but it is also important to emphasize that quality diets such as the Mediterranean diet and DASH diet have been shown to be effective in reducing inflammation and liver steatosis [21,22]. The Moli-sani study observed that adhering to a Mediterranean diet is linked to decreased levels of platelets (PLT) and white blood cells (WBC). These are characteristic inflammatory biomarkers associated with a greater chance of coronary heart disease and cerebrovascular accidents [23]. Although obesity is not linked to greater platelet activation, it is notable that obesity may be connected with a higher PLT count in females with chronic inflammation [24].

The ketogenic diet (KD), with its drastic reduction in carbohydrate intake, is now an internationally recognized weight loss intervention. In particular, a very low-calorie ketogenic diet (VLCKD) is widely accepted as a secure and efficient therapeutic intervention for individuals affected by obesity [25,26,27,28,29]. The rapid mobilization of liver fat and weight loss resulting from VLCKD could represent a useful option for treating MASLD [29,30,31]. Concerning the possible effects of VLCKD itself on inflammation, a reduction in C-reactive protein (CRP) and TNF-α, but not of IL-6, was shown after eight weeks of this diet intervention [32]. In our previous study involving the same dietary treatment of VLCKD for 8 weeks, we found an inhibition of TNF-α and an increase in IL-10, suggesting an inhibitory effect of VLCKD on systemic inflammation with no significant change in IL-6 and IL-8 [33].

The treatment of obesity is being revolutionised by a new class of drugs called glucagon peptide-1 receptor agonists (GLP-1RAs). Recent research has shown that GLP-1RAs play an important role in the regulation of inflammation and the immune system, in addition to controlling glucose homeostasis. In fact, GLP-1RA regulates a number of pro-inflammatory molecular actors, including glucotoxicity, oxidative stress, immune cell recruitment, cytokine production, and lymphotoxicity. GLP-1RA may lessen inflammation either directly through immune cells that express GLP-1 receptors or indirectly through glycaemic control and weight loss [34].

To the best of our knowledge, no information has been previously published about the possible effects of VLCKD on important inflammation parameters such as WBC and PLT counts.

The main objective of this study was to assess the effects of 8 weeks of VLCKD on modifications in WBC and PLT counts and hs-PCR levels, as inflammatory parameters, and on the variations in liver steatosis and fibrosis, analysed by transient elastography (FibroScan) among a group of 87 obese/overweight adults with no obvious comorbidities. Changes in hormone and metabolic biomarkers (insulin triglycerides, total, HDL and LDL-cholesterol, glucose, insulin resistance, uric acid, vitamin D), as well as anthropometric measurements (BMI, waist circumference [WC]) and body composition data (fat mass and fat free mass determined by bioimpedance) were recorded.

## 2. Materials and Methods

### 2.1. Study Design and Population

Our Center of Nutrition for the Research and the Care of Obesity and Metabolic Diseases of the National Institute of Gastroenterology at Saverio De Bellis Research Hospital (Castellana Grotte, Bari, Apulia, Italy) conducted this 8-week real-life prospective study. The fundamental inclusion criteria were age, which had to be between 18 and 64, and a BMI of at least 25 kg/m^2^. An anthropometric assessment, lab tests (biochemistry), and a medical history check were all performed on overweight or obese patients who visited our outpatient clinic. VLCKD contraindications were those listed in National and European guidelines [25,28]. In the medical record survey, the following direct question was adopted to figure out daily alcohol consumption in accordance with American and European daily alcohol consumption recommendations: “Do you drink over two glasses of alcohol per day?” for male patients and “Do you drink over one glass of alcohol per day?” for female patients. This set a threshold of 20 g/day for women and 30 g/day for men. No patient who consumed more than this amount of alcohol was enrolled. At each subsequent session, a physical examination was performed. Additionally, the subjects were asked if they smoked. The local Medical Ethics Committee gave their approval to this study’s protocol (Prot. n. 170/CE De Bellis). This study involved 87 participants and was carried out in accordance with the Helsinki Declaration (1964). Each subject provided written consent just before starting this study. This study’s ClinicalTrials.gov identifier is NCT05477212. From November 2022 to July 2023, overweight or obese patients were enrolled. Three follow-up visits were conducted: at the beginning (T0), three weeks after beginning VLCKD treatment, and eight weeks later (T1). At T0 and T1, data on fasting blood samples and instrumental tests (BIA and Fibroscan) were gathered in addition to anthropometric measurements. This study’s timing has been discussed in previous publications [33,35].

### 2.2. Diet Protocol

The diet protocol used in this study was already published in our previous studies [31,33,34]. The initially two steps were adapted based on Bruci et al.’s description [27]. A VLCKD plan based on a two-step regimen from New Penta, Cuneo, Italy, was followed by all participants. During the two steps, the daily carbohydrate intake was set at 20–50 g, whereas the daily protein intake was fixed at 1–1.4 g/kg of ideal body weight. The recommended daily lipid intake was 15–30 g. A minimum of 2 L of water per day was advised for participants, and they were instructed to consume fewer than 800 Kcal of calories each day. In order to avoid nutritional deficiencies, micronutrient supplements were provided throughout the entire dietary treatment. In the first step, only meal replacements with particular amounts and varieties of vegetables were allowed; in the second step, a protein dish was added to swap out one of the replacement meals.

### 2.3. Anthropometric Parameters

Body height and weight measurements were taken while subjects were fasting, barefoot, wearing light clothing, and with empty bladders to calculate their body mass index (BMI) (kg/m^2^). The same calibrated scale and stadiometer were used to measure each patient. For the purpose of measuring waist circumference (WC), patients had to remove their clothing and stand with their feet close together. The circumference point was located halfway between the lower rib margin and the iliac crest. Using an OMRON M6 automated blood pressure monitor three extemporaneous separate assessments of diastolic (DBP) and systolic (SBP) pressures were taken while the subject was seated. All anthropometric parameters were recorded at baseline, 3 weeks, and 8 weeks after starting VLCKD treatment.

### 2.4. Bioelectrical Impedance Analysis (BIA)

A single-frequency bioimpedance analyser was used for bioelectrical impedance analysis (BIA) (BIA-101 analyser, 50-kHz frequency; Akern Bioresearch, Florence, Italy). All measurements were carried out by an expert nutritionist in line with standardised procedures. According to the recommendations of the European Society of Parenteral and Enteral Nutrition (ESPEN), participants were examined while lying supine with their legs apart [36]. They had spent the 24 h prior to the exam not exercising and the 12 h prior not eating or drinking. After removing socks and shoes, the contact areas were cleaned with alcohol just before placing the electrodes. Previous descriptions of the placement of injector and sensor electrodes (BIATRODES Akern, Florence, Italy) and other BIA applications were accurate [29,36]. Based on the gender, age, weight, and height of each patient, Akern software was used to determine body composition parameters, including validated [37] predictive equations for fat-free mass (FFM, kg) and fat mass (FM, kg).

### 2.5. Biochemistry

After an overnight fast, blood samples were taken between 8:00 and 9:00 in the morning. The automatic haematology analyser Sysmex XT-1000 (Dasit, Cornaredo, Milan, Italy) was used to perform fluorescence flow cytometry to determine the blood cell count. The COBAS 8000 autoanalyzer (ROCHE Diagnostic SPA, Monza, Italy) was used to measure fasting blood glucose, insulin, triglycerides, total cholesterol, LDL-cholesterol, HDL-cholesterol, AST, ALT, gamma-GT, 25-OH Vitamin D, and hs-CRP concentrations. The automatic system for capillary electrophoresis Capillarys 3 OCTA (Sebia Italia S.r.l., Bagno a Ripoli, Firenze, Italy) was used to determine HbA1c. The Homeostasis Model Assessment—Insulin Resistance (HOMA-IR) was used to measure insulin resistance [38].

### 2.6. NAFLD Assessment by FibroScan

The amount of liver fat was measured using the Fibroscan controlled attenuation parameter (CAP) (Echosens, Paris, France), which establishes the level of ultrasound attenuation brought on by hepatic fat at the standard frequency of 3.5 MHz [39]. Studies on obese patients suggest that the diagnostic accuracy of CAP is comparable to that of liver biopsy in the detection of hepatic steatosis. The diagnostic method is increasingly used to quantify fatty liver in patients with suspected hepatic steatosis [40]. While liver fibrosis is present when liver stiffness values are higher than 8.2 kPA, mild MASLD is present when CAP is between 215 and 250 dBm and severe steatosis is present when CAP is higher than 302 dBm [40].

### 2.7. Data Management and Statistical Methods

We performed statistical analysis of baseline variables, expressed as mean ± standard deviation (SD), median, and range for continuous variables. The normality of distribution was assessed for each variable using Shapiro’s test. Statistical significance was determined by the 95% confidence intervals (CI) for *p*-values that were less than or equal to 0.05.

A Generalized Estimating Equation (GEE) [41] was used to estimate the longitudinal trajectories of CAP (pre- and post-VLCKD), liver stiffness (pre- and post-VLCKD), platelet (pre- and post-VLCKD), and WBC count (pre- and post-VLCKD).

The longitudinal trajectories of platelets and WBC (before and post-VLCKD) were estimated using a Generalized Estimating Equation (GEE) between independent groups CAP≤215 (db/m) and CAP > 215 (db/m).

GEE models are helpful for estimating mean changes in biomarker values while adjusting for covariates in biomedical investigations because they allow correlations of response data (repeated measurements on each subject). Due to the non-normal distribution of the outcome variables, a gamma distribution (link identity) was used to model the response, and an unstructured correlation matrix was applied to the data. Age, gender, and smoking habits were all considered as covariates. The results are expressed as mean ± 95% confidence interval (95% CI).

Stata statistical software version 18.0 (StataCorp, 4905 Lakeway Drive, College Station, TX 77845, USA) was used for statistical analysis.

## 3. Results

### 3.1. The Study Population’s Baseline Characteristics and Their Changes following the VLCKD

In the study, 67% of the population were female (N = 58/87), 82% were obese (N = 71/87), and 23% were smokers (N = 20/87). The ages of the participants ranged from 18 to 64, with a mean age of 42.14 (12.90). Table 1 provides an explanation of the anthropometric, metabolic, hormone, and body composition parameters of the study sample both before and after VLCKD.

### 3.2. Changes in Clinical and Laboratory Parameters after the VLCKD

Table 1 displays the major modifications in all parameters following eight weeks of VLCKD. The CAP (the fibroscan parameter of steatosis) and hepatic stiffness (the fibroscan parameter of fibrosis) in the liver were lower after VLCKD. Seemingly, the diet lowered ALT and γGT blood levels. The percentage of subjects with steatosis (CAP > 215 db/m) was 89% (N = 77) before and 62% (N = 54) after VLCKD, while the percentage of subjects with fibrosis (liver Stiffness > 8.2 kPA) was 68.75% (N = 11) before and 31.25% (N = 5) after VLCKD.

After VLCKD, the WBC and platelet counts were significantly reduced. Additionally, BMI, waist circumference, fat mass, fat-free mass, systolic and diastolic blood pressure, insulin resistance (measured by HOMAIR), fasting blood glucose, insulin, HbA1c, triglycerides, total cholesterol, LDL cholesterol, and HDL cholesterol were all decreased following VLCKD. In contrast, after VLCKD, blood levels of vitamin D were higher. VLCKD had no discernible effects on HsCRP (Table 1).

At T1 (after eight weeks of VLCKD), statistically significant decreases were observed for CAP (−47.46, 95% CI −57.58; −37.35), liver stiffness (−0.46, 95% CI −0.91; −0.01), WBC count (−0.72, 95% CI: −0.96; −0.47), and platelets (−24.52, 95% CI: −31.69; −17.35) independently of age, gender, and smoking habit (Table 2). We observed that the decrease in WBCs and platelets with VLCKD was lower in older subjects (−0.95; 95% CI: −1.83; −0.07 −0.95; −0.03 95% CI: −0.05; −0.01, respectively).

The decrease in platelet and WBC counts after eight weeks of VLCKD was statistically significant in patients with a CAP > 215 dB/m (−26.42; 95% CI: −34.13, and −0.85 95% CI: −1.09; −0.60, respectively), whereas subjects without steatosis (CAP < 215 dB/m) did not show a significant decrease in WBC and platelet counts (Table 3).

## 4. Discussion

This study was designed to evaluate the effectiveness of VLCKD in improving the WBC and platelet counts and serum concentrations of hs-CRP, which are representative markers of inflammation, in patients with obesity, a condition typically characterized by low grade inflammation. The investigation was performed on 58 women and 29 men, aged between 18 and 64 years old, with overweight (18%) or obesity (82%), but not taking any drugs.

The research demonstrates a substantial reduction in WBC and platelet counts after 8 weeks of VLCKD (800 kcal/day), but not in hs-CRP serum levels. Since WBCs and platelets are representative markers of inflammation [23], our results demonstrate that VLCKD is followed by a decrease in the typical low grade inflammation that characterizes obesity. This is the first trial to show this effect of VLCKD in patients with obesity. The reduction in platelet numbers is particularly interesting if we take into account that an elevated platelet count is associated with hepatic fibrosis in NAFLD [42]. Furthermore, platelets play a part in the immune system’s activation in addition to taking part in the inflammatory response. In fact, platelets can interact with hyaluronic acid in hepatocytes’ extracellular matrix via the CD44 receptor, leading it to build up in the injured liver, activate T lymphocytes in the liver parenchyma, and ultimately cause hepatic steatosis [43]. Interestingly, the decrease in WBC and platelet counts after the VLCKD was lower in older subjects.

Due to its susceptibility to lifestyle changes, low-grade inflammation has become a crucial target for disease prevention. Intake of polyphenols and foods high in polyphenols has been shown to have positive effects on biomarkers of inflammation (hs-CRP, WBCs, and platelets). In the Moli-sani study, the amount of dietary polyphenols was inversely related to INFLA score, a novel method for examining hs-CRP, WBCs, and platelets as biomarkers of inflammation. To maximise the anti-inflammatory effect, it might be interesting and helpful to add a polyphenol supplement to VLCKD or add polyphenols to meal substitutes [44].

In line with our previous study [30,31], VLCKD was followed by a significant reduction in CAP, the FibroScan parameter measuring the buildup of fatty liver. In a prior study, we proposed that reducing daily carbohydrate intake to less than 50 g, which induces ketosis, would ameliorate NAFLD due to the hepato-protective role of carbohydrate restriction, boosted by ketogenesis and a lower total calorie intake [31]. We cannot exclude the possibility that the effect of VLCKD in lowering steatosis is also mediated by an anti-inflammatory effect of VLCKD per se [30], since a decrease in WBC and platelet counts was obtained. Interestingly, the decreased WBC and platelets count was significant in patients with steatosis, whereas subjects without steatosis (CAP ≤ 215 dB/m) did not show a significant reduction in the number of WBCs and platelets, suggesting that the possible anti-inflammatory effect of VLCKD is higher in patients affected by liver steatosis than in subjects without this condition.

In the present study, VLCKD was also followed by a significant reduction in liver stiffness, the FibroScan parameter quantifying fibrosis liver accumulation. This result was not present in our previous study performed using Fibroscan in a similar population of patients [31]; in our opinion, this result is not surprising if we take into account the fact that 87 patients had been enrolled in the present study versus 33 subjects in our previous study [31].

It is peculiar that, at variance with WBCs and platelets which express cellular inflammation promoting liver fibrosis, CRP circulating levels did not change after VLCKD, suggesting that the possible anti-inflammatory activity of VLCKD is effective on WBC and platelet production, but not on the liver synthesis of hs-CRP, an acute phase protein that may show a non-specific rise within six hours of an inflammatory stimulus. Thus, the changes in WBC and platelet counts seem to be more representative of chronic stimuli acting on WBC and platelet production. Moreover, we cannot exclude that some mechanism associated with VLCKD or to ketone bodies may inhibit the production and/or differentiation of hematopoietic stem cells. Of course, all these hypotheses need to be addressed in specifically designed studies.

This study confirms most of the results reported in our previous study concerning the effects of VLCKD in subjects with obesity [31]. In terms of anthropometric measures, VLCKD substantially lowered WC, fat mass, systolic and diastolic blood pressure, and BMI. VLCKD effectively decreased fasting blood glucose, insulin, insulin resistance (measured by HOMAIR), triglycerides, total cholesterol, LDL cholesterol, HDL cholesterol, and GT in terms of metabolic parameters. The majority of these findings support the idea that VLCKD significantly improves insulin sensitivity [30,31]. In contrast, VLCKD induced an increase in vitamin D levels.

### Strength and Limitations

An important strength of this study is that it is the first to examine the effect of VLCKD in more than 50 subjects, whereas previous studies investigating VLCKD were performed on less than 50 individuals [30]. Additionally, to eliminate any potential interactions, the study was conducted on a group of southern Italian participants with comparable profiles, and it only included people who did not take any pharmacological therapies or supplements. This gives the study methodological legitimacy. We were able to identify the temporal nature of the connections and their linkages because this was a prospective study. FibroScan, which is frequently used in situations of suspected liver steatosis and is the only method suggested by recommendations to assess hepatic steatosis when a biopsy is not feasible, was used to estimate steatosis and fibrosis.

Some limitations must be identified. First of all, this was a real life-study, and was not devised with a control group of patients following a non-ketogenic very low calorie diet; therefore, we cannot state whether the decrease in WBC and platelet counts after VLCKD was due to the low calories, or the ketone bodies, or to yet other causes, and whether the reduction in liver steatosis and fibrosis can be definitely attributed to a decrease in low-grade inflammation.

## 5. Conclusions

We conclude that treating subjects with overweight and obesity by VLCKD is followed by a simultaneous reduction in low-grade inflammation, as demonstrated by a significant decrease in WBCs and platelets, and liver steatosis and fibrosis, as shown by the reduction in CAP and liver stiffness shown by FibroScan. On this basis, we suggest that a decrease in low-grade inflammation may be involved in the improvement of liver health induced by VLCKD. This hypothesis needs to be confirmed by intervention studies showing that a reduction in inflammatory parameters precedes the decrease in liver steatosis and fibrosis levels.

## Figures and Tables

**Table 1:** Description of the whole sample at the assessment time (pre/post-diet). All data are shown as mean (±SD) or median (IQR) or percentage (%).

	Pre-VLCKD	Post-VLCKD	*p*-Value *
	Mean (SD)	Mean (SD)	
N	87	87	
Body Mass Index (kg/m^2^)	35.59 (6.31)	32.59 (6.06)	<0.001
Waist circumference (cm)	112.15 (16.17)	103.98 (15.71)	<0.001
Systolic BP (mmHg)	131.63 (12.42)	123.48 (9.00)	<0.001
Diastolic BP (mmHg)	83.62 (9.16)	76.89 (7.22)	<0.001
FBG (mg/dL)	96.10 (13.12)	88.78 (10.05)	<0.001
Insulin (μU/mL)	16.94 (10.78)	10.25 (6.12)	<0.001
HOMA index	4.10 (3.04)	2.28 (1.47)	<0.001
HbA1c (%)	5.49 (0.52)	5.27 (0.44)	<0.001
Triglycerides (mg/dL)	112.10 (54.79)	89.34 (37.54)	<0.001
Cholesterol (mg/dL)	196.34 (49.57)	168.80 (40.40)	<0.001
HDL Cholesterol (mg/dL)	52.49 (13.82)	46.77 (11.94)	<0.001
LDL Cholesterol (mg/dL)	134.24 (35.51)	113.73 (28.47)	<0.001
25-OH-Vitamin D (ng/mL)	19.78 (6.05)	25.02 (7.11)	<0.001
AST (U/L)	22.33 (10.63)	20.43 (8.71)	0.0617
ALT (U/L)	31.07 (23.71)	25.80 (21.07)	<0.001
γ-GT (U/L)	25.31 (16.16)	16.84 (8.69)	<0.001
CAP (db/m)	287 (255; 325)	230 (188; 278)	<0.001
CAP categories ^§^			
<215 db/m	10 (11%)	33 (38%)	<0.001
≥215 db/m	77 (89%)	54 (62%)	
Liver stiffness (Kpa) ^#^	5.50 (4.30; 6.50)	5.30 (4.00; 6.50)	0.0428
Fat Mass (kg)	40.17 (13.14)	33.41 (11.91)	<0.001
Fat Free Mass (kg)	58.80 (13.27)	56.76 (13.08)	<0.001
hs-CRP	0.42 (0.36)	0.40 (0.34)	0.22
WBC count (10^3^/μL)	6.72 (1.37)	5.98 (1.36)	<0.001
Platelets (10^3^/μL)	269.72 (54.25)	244.91 (58.32)	<0.001
Neutrophils (%)	54.79 (12.06)	56.69 (7.40)	0.26
Lymphocytes (%)	33.00 (7.74)	32.72 (6.41)	0.81
Monocytes (%)	7.67 (1.92)	7.94 (1.86)	0.35
Eosinophils (%)	2.22 (1.53)	2.12 (1.17)	0.67
Basophils (%)	0.57 (0.27)	0.55 (0.25)	0.56
Smoker	54.79 (12.06)	56.69 (7.40)	0.26
Never/Former	67 (77%)		
Current	20 (23%)		

* Signrank tests ^#^ median(IQR) ^§^ Chi square test. Legend: VLCKD: very low-calorie ketogenic diets, BP: blood pressure, FBG: fasting blood glucose, 25-OH-vitamin D: 25-hydroxyvitamin D, AST: aspartate amino transferase, ALT: alanine transaminase, γGT: gamma-glutamyl transpeptidase, CAP: controlled attenuation parameter, hs-CRP: high sensitivity C-reactive protein, WBC: white blood cell.

**Table 2:** Generalized Estimating Equation (GEE): expected values for CAP, liver stiffness, platelet and WBC count by time (pre- and post-VLCKD).

	Post-VLCKD	
	β	95% CI
CAP	−47.46 **	−57.58; −37.35
Liver stiffness	−0.46 *	−0.91; −0.01
Platelets count	−24.52 **	−31.69; −17.35
WBC count	−0.72 **	−0.96; −0.47

Pre-VLCKD referent category. Adjusted for age, gender, and smoking habits. * p value < 0.05 ** p value < 0.001. CAP: controlled attenuation parameter, VLCKD: very low-calorie ketogenic diets, WBC: white blood cell.

**Table 3:** Generalized Estimating Equation (GEE): expected values for platelet and WBC count by time (pre- and post-VLCKD) between two independent groups (CAP ≤ 215 (db/m) and CAP > 215 (db/m)).

	Platelets			WBC Count		
	β	SE	95% CI	β	SE	95% CI
CAP ≤ 215 (db/m)						
Pre-VLCKD	0.00	18.0		0.00	0.33	
Post-VLCKD	2.31		−32.96; 37.57	−0.39		−1.05; 0.27
CAP > 215 (db/m)						
Pre-VLCKD	0.00	3.93		0.00	0.12	
Post-VLCKD	−26.42 **		−34.13; −18.71	−0.85 **		−1.09; −0.60

Adjusted for sex, age, and smoking habits. ** *p*-value < 0.001. CAP: controlled attenuation parameter, VLCKD: very low-calorie ketogenic diets, WBC: white blood cell. SE: Standard Error.

## Data Availability

The original contributions presented in the study are included in the article. Further inquiries can be directed to the corresponding author.
